# The Norwegian PraksisNett: a nationwide practice-based research network with a novel IT infrastructure

**DOI:** 10.1080/02813432.2022.2073966

**Published:** 2022-05-13

**Authors:** Espen Saxhaug Kristoffersen, Bjørn Bjorvatn, Peder Andreas Halvorsen, Stein Nilsen, Guro Haugen Fossum, Egil A. Fors, Pål Jørgensen, Berit Øxnevad-Gundersen, Svein Gjelstad, Johan Gustav Bellika, Jørund Straand, Guri Rørtveit

**Affiliations:** a Department of General Practice, HELSAM, University of Oslo, Oslo, Norway; b Research Unit for General Practice, Department of General Practice, HELSAM, University of Oslo, Oslo, Norway; c Department of Global Public Health and Primary Care, University of Bergen, Bergen, Norway; d Department of Community Medicine, UiT – the Arctic University of Norway, Tromsø, Norway; e Research Unit for General Practice, NORCE Norwegian Research Centre, Bergen, Norway; f Research Unit for General Practice, Department of Public Health and Nursing, Faculty of Medicine and Health Sciences, Norwegian University of Science and Technology, Trondheim, Norway; g Medrave Software AS, Tønsberg, Norway; h Norwegian Centre for E-health Research, University Hospital of North Norway, Tromsø, Norway

**Keywords:** Clinical interventions, family practice, health services research, quality development, primary care, general practice, practice-based research networks

## Abstract

**Purpose:**

Clinical research in primary care is relatively scarce. Practice-based research networks (PBRNs) are research infrastructures to overcome hurdles associated with conducting studies in primary care. In Norway, almost all 5.4 million inhabitants have access to a general practitioner (GP) through a patient-list system. This gives opportunity for a PBRN with reliable information about the general population. The aim of the current paper is to describe the establishment, organization and function of PraksisNett (the Norwegian Primary Care Research Network).

**Materials and Methods:**

We describe the development, funding and logistics of PraksisNett as a nationwide PBRN.

**Results:**

PraksisNett received funding from the Research Council of Norway for an establishment period of five years (2018–2022). It is comprised of two parts; a human infrastructure (employees, including academic GPs) organized as four regional nodes and a coordinating node and an IT infrastructure comprised by the Snow system in conjunction with the Medrave M4 system. The core of the infrastructure is the 92 general practices that are contractually linked to PraksisNett. These include 492 GPs, serving almost 520,000 patients. Practices were recruited during 2019–2020 and comprise a representative mix of rural and urban settings spread throughout all regions of Norway.

**Conclusion:**

Norway has established a nationwide PBRN to reduce hurdles for conducting clinical studies in primary care. Improved infrastructure for clinical studies in primary care is expected to increase the attractiveness for studies on the management of disorders and diseases in primary care and facilitate international research collaboration. This will benefit both patients, GPs and society in terms of improved quality of care.

**Key points**
- Practice-based research networks (PBRNs) are research infrastructures to overcome hurdles associated with conducting studies in primary care
- Improved infrastructure for clinical studies in primary care is expected to increase the attractiveness for studies on the management of disorders and diseases in primary care and facilitate international research collaboration
- We describe PraksisNett, a Norwegian PBRN consisting of 92 general practices including 492 GPs, serving almost 520,000 patients
- An advanced and secure IT infrastructure connects the general practices to PraksisNett and makes it possible to identify and recruit patients in a novel way, as well as reuse clinical data
- PraksisNett will benefit both patients, GPs and society in terms of improved quality of care
- This paper may inform and inspire initiatives to establish PBRNs elsewhere

## Introduction

Countries with strong primary care systems have better health outcomes and a more cost-effective health care [1,2]. In Norway, as many as 69% of the entire population visit their general practitioner (GP) over a one-year period [3]. This amounts to about 15 million GP visits per year. A multitude of health problems are diagnosed and managed in primary care. Clinical research tailored to reduce knowledge gaps in primary care is a prerequisite for improved quality of care in the whole health care system. Paradoxically, very few clinical trials are conducted in primary care in Norway. Most clinical guidelines are therefore based on studies of patients seen in hospital settings.

Barriers for GPs to be involved in research projects include uncertainty about the relevance of the research question, feasibility of project workload, regulatory approvals and possible risks for the practice or patients. The latter is also related to IT tools interfering with the electronic health record (EHR) system [4–7].

A major obstacle for primary care research is the absence of an infrastructure to reduce such barriers and support the identification and inclusion of patients as well as obtainment of high quality data [8]. These are cumbersome processes, particularly in a primary care research context. First, each GP needs to be recruited for the study. Second, the GPs and the researchers must identify, recruit and follow-up patients in the primary care population, and data must be accessed and handled accordingly. Even studies on prevalent conditions will need recruitment from multiple general practices, further complicating matters.

Norwegian primary care research institutions have the ambition, capacity and competence to access patient data [9] and to conduct randomized clinical trials (RCTs) [10–14] in spite of logistic barriers. However, up until now, there has been no research infrastructure available to facilitate clinical studies or otherwise access patients or patient data for research in primary health care.

Internationally, practice-based research networks (PBRNs) have been successfully set up in the UK, Netherlands, USA, Ireland, Canada and Australia [15–21], and they have recruited patients to produce high-quality clinical research [22–26]. Even if the main purpose of the PBRNs is to facilitate clinical research, important beneficial side effects of the research are quality improvement and implementation of research-based knowledge [27–30]. Internationally, the scientific output from PBRN-based research is high and clearly unique due to valid, high-quality data from the relevant context [22–25,31,32].

In Norway, the Research Council of Norway (RCN) has supported the establishment of the Norwegian Primary Care Research Network (PraksisNett) over a five-year period, starting in January 2018. The aim of the current paper is to describe the establishment, organization and function of PraksisNett. The paper may inform and inspire initiatives to establish PBRNs elsewhere and may function as a methods reference for upcoming studies using the network.

## Methods

### Setting

Norway has roughly 5.4 million inhabitants. Provision of primary health care is organized at the municipality level where most GPs work in private enterprises based on a contract with the municipality. The service is organized as a patient-list system. Each Norwegian citizen has through legislation the right to be enlisted with a GP. In 2019, the average list per GP included 1068 patients and more than 99.8% of population were enrolled in the scheme [33]. Group practices of 3–6 GPs with shared responsibility, a low level of administrative management and a few medical secretaries is the most common organization locally. Income for GPs comes from a combination of a fixed annual fee per listed patient from the National Health Insurance, remuneration for consultations from the state and a small co-payment from patients (up to an annual limit of around 271 Euro including all public health care services and medications). In some districts, GPs are employed with fixed salary by the municipality. Norway has a universal healthcare system, and Norwegian GPs are gatekeepers for admission to specialists and hospitals except in emergencies. Norwegian hospitals are almost exclusively publicly financed, with a mixture of smaller primary hospitals and larger university hospitals.

### The early planning phase

The process of establishing the Norwegian PBRN started in 2010 with a letter to the Ministry of Health, stating that clinical research in primary care were lagging behind in comparison to research in secondary care, partly due to lack of an infrastructure for research. The initiative was a collaboration between academic GPs and academic dentists. The letter referred to the international experience with successful PBRNs and urged the Ministry to establish corresponding networks in Norway while highlighted that funding was a major barrier and the main reason why Norway was without a PBRN. This resulted in seed funding from the Directorate of Health, and a working group was established. Study trips were arranged to Bristol (England), Dundee (Scotland), Amsterdam (the Netherlands) and later Toronto (Canada). Reports were made, and pilot studies to test different aspects of the future network were set up [34]. The international networking also resulted in workshops at international conferences and cross-country participation in advisory boards. A first joint grant proposal from academic GPs and dentists to the RCN in 2014, received encouraging feedback but no funding. In 2015, the government decided to establish a PBRN for research in dentistry care only, which unfortunately has not yet been established. Based on the urgent need for more clinically relevant research, experiences from previous workshops, input from international academic GP partners and a strong belief that Norwegian primary care need a PBRN, the work to establish PraksisNett continued. Academic GPs therefore successfully applied for funding from the RCN in 2016 (see further below).

## Results

In this section, we describe the establishment of the research infrastructure PraksisNett – the Norwegian Primary Care Research Network.

### Aims of PraksisNett

The vision for PraksisNett is to expand the knowledge base for GPs, in order to improve diagnosis, treatment and follow-up for patients in the community. By facilitating the identification and inclusion of eligible patients for clinical trials, PraksisNett aims to substantially

- increase both quality and quantity of clinical research projects in primary care
- allow Norwegian patients, clinicians and researchers to be involved in excellent, clinical research
- enhance active collaboration with internationally leading primary care research environments
- share knowledge, expertise and best practice within primary care

### Description of the PraksisNett organization

The participating general practices represent the core of PraksisNett and are further described below. The PraksisNett organization itself is based on two interdependent parts: (i) a human resource-based infrastructure and (ii) an advanced, secured IT infrastructure connecting the general practices to PraksisNett.

**Figure 1.**
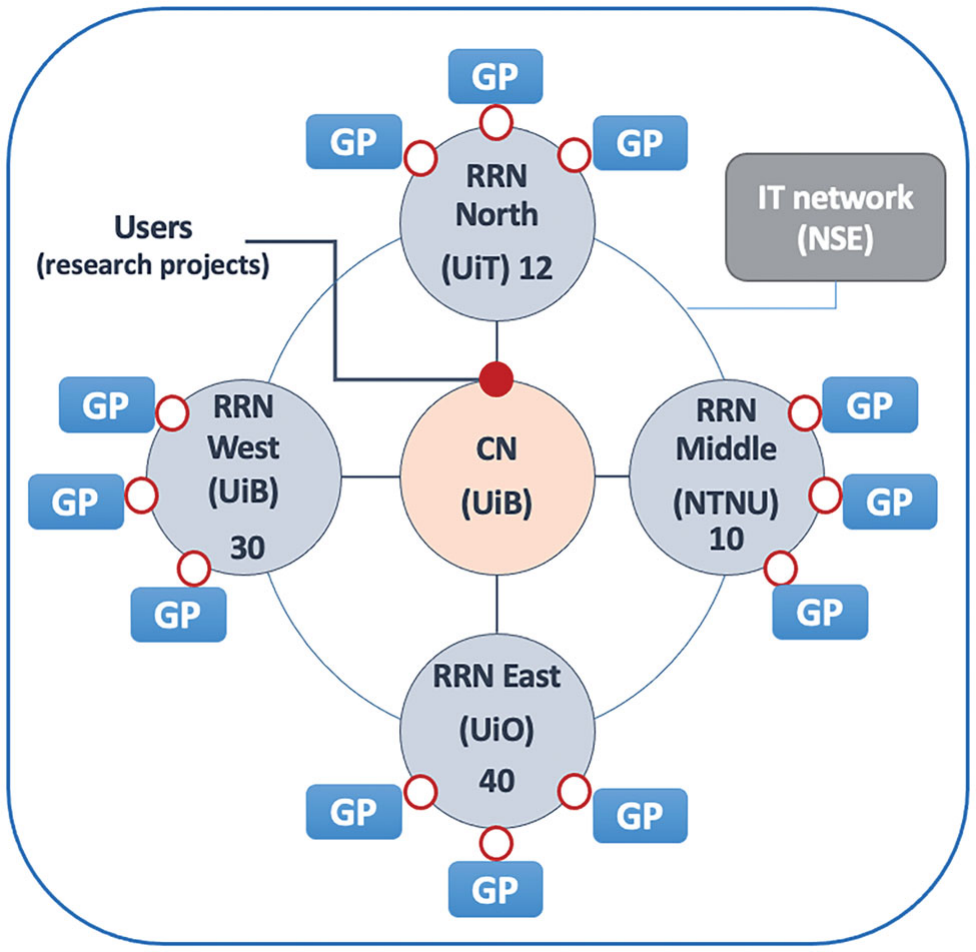
Structure of the Norwegian practice-based research network (PBRN). CN: coordinating node; RRN: regional research network; GP: general practice; UiB: University of Bergen; UiO: University of Oslo; UiT: University of Tromsø – the Arctic University of Norway; NTNU: Norwegian University of Science and Technology; NSE: Norwegian Centre for E-health Research. Numbers indicate the number of participating practices within each RRN. An underlying IT infrastructure interconnects GPs and RRNs, sharing tools and background data extraction mechanisms. (small open circles). Users are able to obtain research data from GPs via the IT infrastructure (small black circle).

- The human resource-based infrastructure consists of four executive regional research networks (RRNs) located at the medical faculties of the Universities of Oslo, Tromsø and Trondheim, as well as the NORCE research institute in Bergen. The RRNs are interconnected with the coordinating node (CN) located at the medical faculty of the University of Bergen (Figure 1). The CN is the entry point for users/researchers and is responsible for strategy and outward communication for the network, as well as contact with sponsors and collaborators. The RRNs have the operative responsibility for the participating general practices and provide support to researchers and clinicians. Each RRN engages 10–40 general practices. The RRNs provide feasibility assessment, access to study design tools, assist in service support cost estimation, make a recruitment plan with the researchers and assist researchers with interaction with and training of the GPs.
- PraksisNett has adopted an IT infrastructure, called Snow, named after the founder of epidemiology, John Snow (1813–1858) with an architecture similar to the PORTAL clinical data research network, OHDSI and PCORnet [35–37]. Importantly, in the Snow system, no central database is required, as each general practice stores data within its own IT infrastructure, warranting local control of processing and ensuring the anonymity of sensitive patient information [38–40]. Privacy by design principle, extensive risk assessment and risk mitigation measures have been applied to reduce the risks [39,40]. As the Snow system only handles pseudonymized and encrypted patient data the risks from using the system, related to being a data controller (General Data Protection Regulation (GDPR) responsibility) is minimized. The technology allows online distributed analyses of the data repositories for the researchers to plan studies, for the network to perform basic statistics and for the GPs to locally generate recruitment lists of eligible patients. Other researchers have shown that it is feasible to reuse clinical data from electronic health record (EHR) to recruit patients to clinical research [38]. The Snow system can perform the basic statistical analyses for power calculations. The local internal storage also supports practice-internal quality improvement work [39]. Data extraction tools and software are a part of the Snow system, which has been operational since 2010 [40]. The IT infrastructure uses the Substitutable Medical Applications and Reusable Technologies (SMART) on Fast Healthcare Interoperability Resources (FHIR) bulk data Application Programming Interface (API) for standards-based data extraction from EHR systems. This component is installed on the EHR servers. It uses data extraction tools developed through longstanding collaboration with Medrave Software AS to extract data from most of the general practice EHR system vendors in Norway. This data reuse component (DRC) ensures that the electronic data use agreements are fulfilled and thereby easily facilitates access to EHR data for studies when digitally signed by the GPs. The Medrave M4 software is designed for reusing EHR data in intuitive reports for the GPs’ to gain increased insight in their own practice and with the possibility to drilldown in graphs and tables for identification of patients that may be in need of follow-up. Furthermore, the software includes benchmark modules for easy and anonymous comparison of own practice compared to other GPs and with the possibility to follow indicators over time as part of quality assurance work. In collaboration with Center for Quality Improvement in Medical Practices, Medrave Software has developed several reports and made them available to the participants of PraksisNett for use in CME group courses.

### Partners

The partners responsible for the RCN grant application and the establishment of PraksisNett are the academic primary care units at the four medical faculties in Norway, i.e. at the University of Oslo, University of Bergen, University of Tromsø—the Arctic University of Norway and the Norwegian University of Science and Technology in Trondheim, together with the research institute NORCE and the Norwegian Centre for E-health Research at University Hospital of Northern Norway.

### PraksisNett management

Operational-level management is ensuring daily operation of the nodes (CN, RRNs). The Management Board consists of the project leader and the leaders of the three work packages (WPs) (WP1: CN; WP2: the regional networks including recruiting and retaining practices; WP3 the IT infrastructure), CN and the RRNs and is responsible for the overall strategical and operational management of PraksisNett and for the implementation of the infrastructure. The Steering Committee is comprised by the deans of each medical faculty and research directors at NORCE and the Norwegian Centre for E-health Research. An International Advisory Board with four distinguished academic primary care leaders, and a National Advisory Board representing stakeholders in Norway, give advice to the Steering Committee and the Management Board.

### Recruitment of general practices

**Figure 2.**
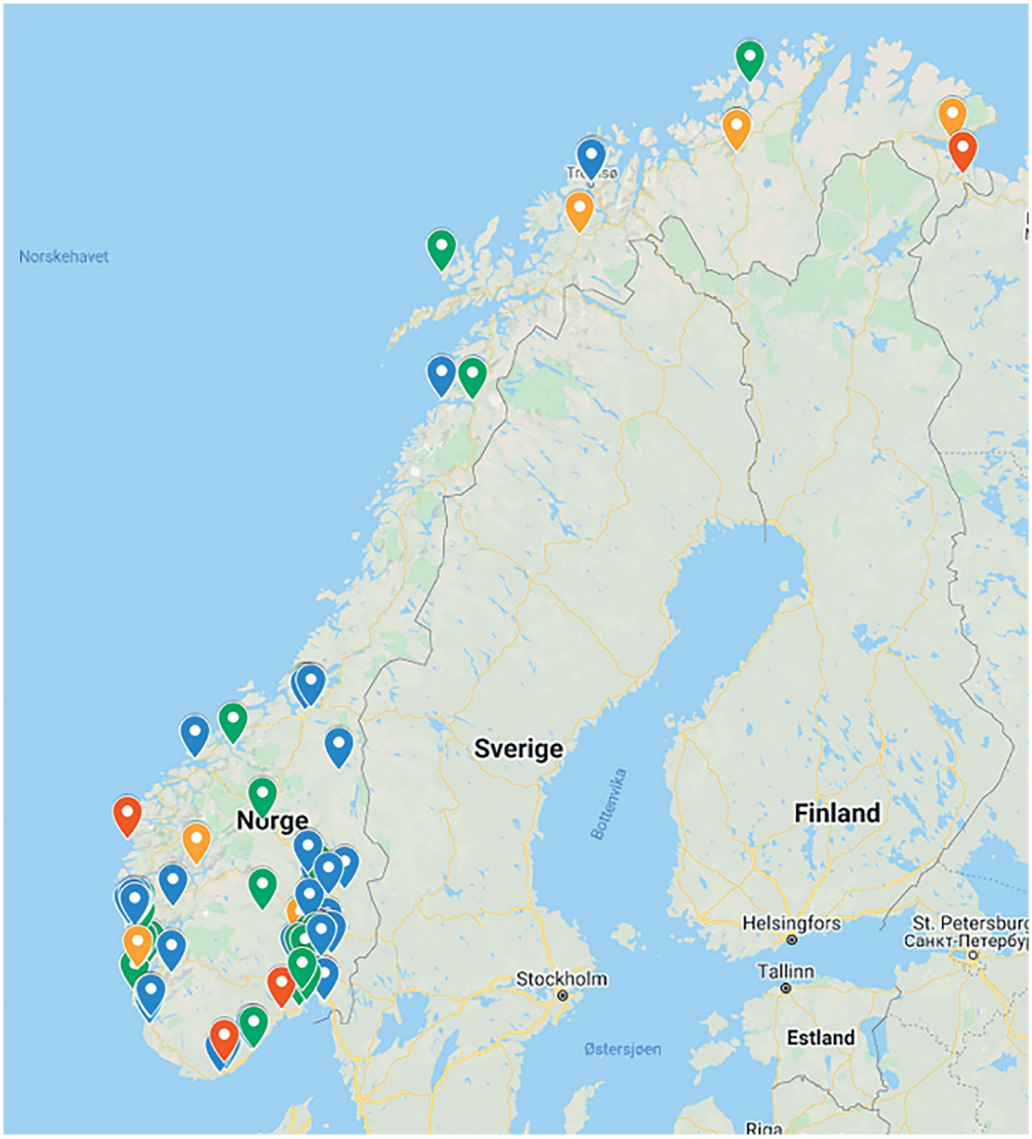
The 92 general practices recruited to PraksisNett. Green denotes 2–4 GPs per general practice, blue 5–7 GPs, yellow 8–10 GPs and red > 10 GPs (map from google.com/maps).

The current funding allows the recruitment of 90 practices all over Norway. Practices were recruited during 2019–2020. A plan for how to recruit the practices to ensure reasonable representativeness was made prior to the recruitment process. The inclusion criteria were set to >2 GPs or >3000 patients enlisted with the practice. Additionally, all GPs in the practice should accept sharing aggregated data, and the practice must participate in at least one study per year. Altogether, the practices had to have a representative geographical distribution with both rural and urban practices. GPs were informed about PraksisNett at national meetings, courses, clinical and academic networks, social media, articles in the Norwegian GP journal (‘Utposten’) and the Journal of the Norwegian Medical Association [41,42]. Interested general practices (*N* = 150) completed a short web-based questionnaire, among which 126 fulfilled the inclusion criteria. Most of these (*n* = 121) were invited to an information meeting at their office. Fifteen practices declined participation after the information meeting. The main reasons for non-participation were registered and included fear of too much work, a feeling of recruiting patients without being actively involved in the research, planned changes in own EHR system, changes in the working staff and not interested in research. Six other practices were interested after the information meeting but asked to be placed on a waiting-list temporarily. Eight practices did not respond to the meeting invitation and received no more detailed information about PraksisNett. In total, 92 practices were recruited to PraksisNett: 40 practices from region East, 30 from West, 10 from Middle and 12 from North (Table 1 and Figure 2). The practices were recruited sequentially in different regions, according to planned capacity of the network. Formal contracts were signed by the RRNs and each of the 92 practices.

**Table 1.** Descriptive data of the general practices participating in PraksisNett by geographical area.

	Total	East	West	Middle	North	Norway [33]
General practices (n)	92	40	30	10	12	1374
No. of GPs per practice, mean (min–max)	5 (2–17)	5 (3–13)	6 (2–17)	5 (3–7)	6 (3–11)	NA
GPs (n)	492	199	168	52	73	4951
Age of GPs (years), mean (min–max)	47.5 (29–73)	49.2 (30–73)	46.1 (29–71)	47.0 (30–69)	46.8 (29–68)	47.2 (28–75)
Sex, % (n)						
Female	48.4 (238)	48.2 (96)	45.8 (77)	48.1 (25)	54.8 (40)	45.8
Male	51.6 (254)	51.8 (103)	54.2 (91)	51.9 (27)	45.2 (33)	54.2
Specialist of general practice, % (n)	68.3 (336)	75.9 (151)	60.1 (101)	75.0 (39)	61.6 (45)	63.2
Years as physician, mean (SD)	17.2 (10.7)	18.7 (10.9)	16.2 (11.0)	16.9 (10.1)	15.8 (9.6)	NA
No. of list-patients, mean (min–max)	1057	1122	1078	1052	837	1068
	(295–2260)	(295–2260)	(300–2010)	(400–1450)	(300–1500)	(100–2500)

NA: not available; GP: general practitioner.

These 92 practices include 492 GPs, serving almost 520,000 patients. This corresponds to 10% of Norwegian GPs and approximately 9.5% of the total Norwegian population. The practices are distributed all over the country, they are a mix of rural and urban practices and we consider them to be reasonable representative for Norwegian general practices in terms of age, gender, urban vs. rural distribution and number of patients on GP-list (Table 1 and Figure 2).

### Use of patient data

The infrastructure provides access to aggregated anonymous routine data directly from the general practices. Aggregated anonymous routine data include information about the GPs, the patients’ medical history, demographics, number of contacts, diagnosis, sick-leave, referrals, laboratory testing and prescriptions. Such data are also used for administrative and research purposes within PraksisNett and can be used to calculate number of eligible patients within the network and to compare for differences in recruited vs eligible patients. Additionally, individual data that have been obtained routinely can be extracted and used in research projects after ethical approval and consent from GPs and/or the patients. Different types of such retrospective data may be available based on the different EHRs. Finally, data that need to be collected prospectively and solely for the purpose of a particular research project may be obtained according to ethical approval and informed consent from patients.

### Integrating the IT infrastructure with the practices

When fully operative, the Snow infrastructure will increase the performance of PraksisNett considerably, also compared to many international PBRNs, as PraksisNett will have the IT infrastructure necessary to plan studies, identify and invite patients and monitor routine and clinical data for studies in primary care. This is a novel and innovative part of PraksisNett but also the part that has demanded most resources and caused delays in the establishment of the infrastructure. Especially, the collaboration with many different EHR vendors, changes in EHR hosting from local servers to web-based servers and the introduction of GDPR have so far caused several time-consuming delays and adjustments during the establishment period.

### Financial compensation for participating general practices

The practices receive financial compensation for their time and efforts: All practices receive a fixed annual compensation, corresponding to about 2000 Euros, which covers time spent on communication with PraksisNett, including consideration of research project participation etc. In addition, each GP receives service support cost based on actual participation and time used in each research project. The service support costs are decided by the Management Board prior to each study based on calculations on expected time use. This time should cover study set up in the practice, data search and procedures for contacting patients and actual work relating to obtaining informed consent, inclusion, investigation of the patients and data registration.

### Selecting research projects using PraksisNett

Up-to-date information about how to use PraksisNett as a researcher, the costs and all the necessary requirements to submit an application are easily available on the webpage https://www.uib.no/en/praksisnett.

In short, the process starts when a project leader makes initial contact with PraksisNett through the webpage or directly to one of the RRNs leaders. Informal feedback and advice are given on early project schemes and includes guidance to the principal investigators about how to use PraksisNett efficiently. When projects are ready for full assessment, the principal investigator is encouraged to send a formal application to PraksisNett. The formal application should include the protocol, study information to the GPs and the patients, informed consent, budget, data management plan, approval from the local Research Ethics Committee, the Data Protection Impact Assessment and the evaluation by the Data Protection Officer. The selection process to identify eligible research projects is handled by the PraksisNett Management Board. Evaluation criteria include the scientific quality, the clinical relevance of the study for primary care and the ethical and technical feasibility. Both early project schemes and complete applications may be discussed several times in the Management Board to ensure that all relevant information are in place before a final decision is reached. In approved projects, PraksisNett and the project’s host institution agrees on a signed contract. By January 2022, several studies have used PraksisNett to recruit GPs and patients in research (Table 2).

**Table 2.** Examples of studies currently using PraksisNett.

Topic	Recruitment	Design
Upper respiratory tract infections	GPs	AUDIT, questionnaire
Sinusitis	GPs and patients	Qualitative
COVID-19	GPs	AUDIT, questionnaire
Depression in young adults	GPs and patients	Qualitative
Laboratory use	Aggregated data	Data extraction study
Sinusitis	GPs	Data extraction study
Shoulder pain	GPs	Cluster randomized
Hypertension	GPs and patients	Observational cohort
Hearing and balance in elderly	GPs and patients	Cross-sectional
Sciatica	GPs and patients	Observational cohort and recruitment to randomized controlled drug trial
Osteoarthritis	GPs	Data extraction study
Sinusitis	GPs and patients	Randomized controlled drug trial
Maternity care	GPs and patients	Questionnaire
Sleep and infections	GPs and patients	Data extraction and questionnaire
Medically unexplained physical symptoms	GPs and patients	Qualitative
Insomnia	GPs and patients	Randomized controlled trial

GP: general practitioner.

PraksisNett aims toward a mix of pharmacological and non-pharmacological RCTs, other interventional studies, cross-sectional and prospective observational studies, data extraction studies, AUDITs and qualitative studies.

Although PraksisNett aims to stimulate academic GPs to use the network for own research and international collaboration, PraksisNett as an infrastructure organization does not in itself initiate research projects. However, the management team comprises GP academics, who may initiate research projects using PraksisNett on the same conditions as other researchers.

### Ethics and data security

All research projects intending to use PraksisNett will have to apply for approval from the local Research Ethics Committee and get an evaluation by the Data Protection Officer before the study is approved by the Management Board. Each principal investigator is responsible for their own project which includes secure management of the research data, and ensuring that all ethical, privacy and data security requirements are fulfilled. The use of aggregated (i.e. anonymized) data does not need ethical approval or informed consent from individual patients. In Norway,provided that the Research Ethics Committee gives dispensation, individual patient data for use in data extraction studies may not require informed consent in special circumstances, according to the Health Personnel Act §29, the Health Register Act §19e and the Public Administration Act §13d. However, the use of such data is regulated by the written contract between PraksisNett and the GPs.

PraksisNett is committed to patient safety and the highest ethical standards and will employ only approved procedures and tools, complying with national and international laws and regulations. Patients may decline participation in clinical studies or contribution of individual data at any time up to the point of data extraction and transfer of research datasets to a safe haven service. Patients participating in research will have the possibility to be informed about how their data are used according to the GDPR. Patients can also withdraw their consent to participation, as required to comply with GDPR. The overall data management plan and the Data Protection Impact Assessment for PraksisNett are recommended by the Data Protection Officer at the University Hospital of Northern Norway.

## Discussion

Undertaking clinical studies in general practice effectively requires logistics which in most cases are expensive, time-consuming and labor-intensive. The lack of relevant clinical research contributes to substantial knowledge gaps and inhibits innovation, education and quality improvement in primary care. This in turn has negative consequences for patients due to suboptimal quality of care. PraksisNett addresses these challenges by facilitating clinical studies which can test effects of new, innovative strategies for diagnosis and management of health problems and diseases mainly managed in primary care, including prevalent disorders like obstructive lung disease, diabetes, anxiety, depression, pain, migraine, sleep problems, infections and musculoskeletal disorders [43]. Although still in the establishment phase, we believe that PraksisNett with its secure IT infrastructure and high trust among Norwegian GPs will contribute to substantially increase quality and quantity of clinical studies in primary care and make Norwegian primary care attractive for multinational studies.

PraksisNett also has potential to be extended to the full range of primary care clinics, including physiotherapists, out-of-hours services, dentists, nursing homes etc.

The GPs participating in PraksisNett have a similar profile as GPs nationally, except a slightly higher proportion of female GPs and somewhat higher proportion of certified specialists of general practice [33]. Although some of the participating GPs may be a selected group of research-interested clinicians, it is reasonable to assume that their patient population (almost 10% of inhabitants in Norway) is representative of the Norwegian population. When the IT infrastructure is fully operable, numbers from PraksisNett will be compared to those from Statistics Norway. The representativeness and the opportunity to compare recruited patients with eligible patients in each study are major strengths of PraksisNett.

The recruitment of GPs to PraksisNett was surprisingly straightforward. We think one reason is that PraksisNett is managed by a group of experienced primary care researchers, some still working part time as GPs and a good standing within the GP community. In-depth knowledge of the field and a strong focus on feasibility and minimizing barriers in everyday practice are known to be important factors when recruiting GPs [4,5,7]. A qualitative study among Norwegian GPs prior to the establishment of PraksisNett also showed that the most important incentives for participation in PBRNs were participation in clinically relevant research projects made feasible within a busy everyday practice. Furthermore, all formal approvals and research organizing must be handled by others than the GPs. The GPs highlighted that the administrative part of the PBRN must be robust, predictable and with access to resources to solve practical problems immediately. Income was not seen as a major incentive, but it was important that the GPs were compensated in one way or another. Software supporting internal quality improvement was also deemed positive [44].

Thus, the possibility of quality improvement in the practices and better access to academic detailing based on findings from studies conducted through PraksisNett seem to be attractive for the practices. This includes free access to the Medrave M4 software which is a well-known and well-reputed EHR data extraction system for many Norwegian GPs.

As PraksisNett is still under development, the first studies (Table 2) have been carefully chosen to pilot different parts of the infrastructure and still supporting the purpose of the proposed studies. Particularly, integrating the Snow system with the practices has been demanding and needed tailor-suited piloting. Innovation and development within EHRs are welcome but further challenges the integration. Whereas some researchers have used the Medrave M4 data extraction tool to access data, none of the studies have yet used the full version of the PraksisNett data infrastructure. Within the establishment period ending December 2022, PraksisNett aims to use a fully operable IT infrastructure in RCTs.

Based on the planning and the establishment of PraksisNett, we believe this will prove to be an important research infrastructure in Norwegian primary care which will improve quality and quantity of primary care research with high societal gains. However, it is the experience of other PBRNs as well as ours, that predictable long-term funding is crucial to develop and maintain a robust infrastructure for primary care research. We believe that our success in getting the competitive NRC grant lies particularly with three factors: first, long-term, solid planning including agreement and support from all the academic general practice units and GP associations in Norway; second, international support from experienced research environments and strong researchers and third, the systematic piloting of the planned project prior to the application.

The PraksisNett infrastructure is expensive and must prove itself worthwhile by obtaining the ambitious goals for enhanced quality and quantity of clinical research projects. This is a long-term goal that implies funding longer than the establishment period of just five years. We are currently working to secure a more stable public funding from year 2023 onwards, and the ambition is to secure funding over the state budget.

## Conclusion

Norway has established a nationwide PBRN, PraksisNett, to increase the number, quality and output of clinical research projects through facilitating inclusion of primary care patients in research. A predictable long-term funding will be crucial to maintain and further develop the infrastructure. PraksisNett will benefit patients, primary care and the society in terms of improved quality of care.
